# Gender influences left ventricular remodelling in the setting of aortic stenosis but does not appear to impact on reverse remodelling following transcatheter aortic valve implantation

**DOI:** 10.1186/1532-429X-17-S1-P332

**Published:** 2015-02-03

**Authors:** Laura E Dobson, Tarique A Musa, Akhlaque Uddin, Timothy A Fairbairn, Daniel J Blackman, David P Ripley, Adam K McDiarmid, Peter P Swoboda, Bara Erhayiem, Pankaj Garg, Sven Plein, John P Greenwood

**Affiliations:** 1 Multidisciplinary Cardiovascular Reseacrh Centre & Leeds Institute for Cardiovascular and Metabolic Medicine, University of Leeds, Leeds, UK; 2 Yorkshire Heart Centre, Leeds Teaching Hospitals Trust, Leeds, UK

## Background

Left ventricular (LV) remodelling in the setting of the chronic pressure overload of aortic stenosis (AS) appears to differ according to gender. Women appear to have an improved survival compared to men following transcatheter aortic valve implantation (TAVI) but the reasons for this are yet to be established. Potential mechanisms include differing patterns of LV reverse remodelling, LV fibrosis regression and reduced aortic regurgitation in females. We sought to establish using cardiac magnetic resonance (CMR) imaging, the reference standard non-invasive technique for LV mass and scar quantification, whether there were any gender differences in these parameters before and after TAVI.

## Methods

53 patients with symptomatic severe aortic stenosis undergoing TAVI were prospectively enrolled between April 2009 and March 2014. Patients with contraindications to CMR were excluded and all patients provided informed written consent. All patients underwent an identical 1.5T CMR protocol (Intera, Philips) prior to and at a median of 6 months following TAVI (IQR 5-6 months). Multi slice, multiphase imaging was carried out using a standard steady-state free procession pulse sequence in the short axis to cover the entire left ventricle. Late gadolinium enhancement imaging was performed 10min after the administration of 0.2mmol/kg of gadoteric acid (Doteram, Guerbet, Villepinte). Quantitative analysis was performed using dedicated computer software (CVI^42^, Circle Cardiovascular Imaging, Alberta, Canada).

## Results

Basic clinical, echocardiographic and procedural characteristics can be seen in Table 1. Women with severe AS have a smaller indexed LV mass (LVMi) (69.6 ± 18.8g/m^2^ Vs 81.8 ± 21.6g/m^2^,p=0.03) less baseline myocardial scar (1.7±2.5g Vs 5.0 ± 5.7g, p=0.01) and a smaller indexed LV end diastolic volume (LVEDVi) than men (90.2 ± 17ml/m^2^ Vs. 105 ± 27.7 ml/m^2^;p=0.03). A trend towards a smaller indexed left atrial volume (LAVi) (63.7 ± 17.2ml/m^2^ Vs 74.2 ± 23.5ml/m^2^, p=0.08) and better ejection fraction (57.9 ± 11.4% Vs 52.6 ± 13.8%, p=0.15) was also seen in women when compared with men. Six months following TAVI, there was no significant difference seen between genders with regards to LV mass regression, change in ejection fraction, change in myocardial scar burden, change in left atrial volume or change in aortic regurgitant fraction (Table 2).

**Table 1:** Basic clinical, echocardiographic and procedural characteristics. Data expressed as mean ± SD unless otherwise stated.

	Male	Female
Number of patients, (%)	30 (57)	43

Age	78 ± 6	84 ± 8

STS Mortality score, %	4.2 ± 2.4	7.2 ± 3.6

STS Morbidity/mortality score, %	23.8 ± 8.1	28.1 ± 8.8

Hypertension, n (%)	10 (33)	14 (61)

Echocardiographic characteristics		

AVAi (cm/m2)	0.32 ± 0.1	0.32 ± 0.08

Peak pressure drop	86.3 ± 22	93.8 ± 21.6

Procedural characteristics		

TAVI Type, n (%)		

Medtronic Corevalve	22 (73)	19 (83)

Boston Lotus	8(27)	3 (13)

Medtronic Engager	0 (0)	1 (4)

TAVI size, n (%)		

23mm	2 (7)	4 (17)

26mm	2 (7)	7(30)

27mm	7 (23)	1 (4)

29 mm	17 (56)	11 (49)

31mm	2 (7)	0 (0)

Access site, n (%)		

Femoral	26 (65)	21 (92)

Subclavian	3 (10)	1 (4)

Apical	0 (0)	1 (4)

Direct aortic	1 (4)	0 (0)

STS: Society of Thoracic Surgeons

**Table 2:** Pre and 6 month post-TAVI values for male and female gender. Values expressed as mean ± SD.

	Pre-TAVI	Post-TAVI	Change	P Value
LV Ejection fraction (%)				

Men	52.6 ± 13.8	54.7 ± 11.3	2.1 ± 8.1	0.18

Women	57.9 ± 11.4	59.5 ± 11.8	1.5 ± 6.5	0.28

P Value	0.15	0.15	0.79	

LV End diastolic volume indexed (ml/m2)				

Men	105.0 ± 27.7	97.2 ± 21.9	7.8 ± 20	0.04

Women	90.2 ± 17.0	81.8 ± 19.3	8.4 ± 15.8	0.02

P Value	0.03	0.01	0.92	

LV Mass indexed (g/m2)				

Men	81.8 ± 21.6	64.0 ± 17.9	17.7 ± 9.1	<0.001

Women	69.6 ± 18.8	53.9 ± 14.4	15.7 ± 8.4	<0.001

P Value	0.04	0.03	0.43	

LV scar (g) (FWHM method)				

Men	5.0 ± 5.7	5.1 ± 6.2	0.1 ± 2.1	0.88

Women	1.7 ± 2.5	1.3 ± 2.6	0.6 ± 1.4	0.25

P Value	0.01	0.01	0.36	

Left atrial volume indexed (ml/m2)				

Men	74.2 ± 23.5	65.5 ± 23.3	8.7 ± 13.7	0.002

Women	63.7 ± 17.2	58.3 ± 19.3	5.4 ± 31.0	0.2

P Value	0.08	0.25	0.47	

Aortic regurgitant fraction (%)				

Men	12.8 ± 8.2	6.5 ± 6.5	6.3 ± 9.3	0.001

Women	9.7 ± 9.5	6.2 ± 5.2	3.0 ± 10.5	0.12

P Value	0.22	0.87	0.25	

FWHM: Full width half max

## Conclusions

In the setting of the chronic pressure overload of aortic stenosis, there appears to be a difference in LV remodelling between genders with a smaller LV cavity, less fibrosis and a lower LV mass in women compared with men. Six months following TAVI, there did not appear to be a significant difference in LV reverse remodelling, change in myocardial scar burden or aortic regurgitant fraction according to gender.

## Funding

This study was part funded by the British Heart Foundation (BHF) (PG/11/126/29321).

